# Construction of a diagnostic model for temporal lobe epilepsy using interpretable deep learning: disease-associated markers identification

**DOI:** 10.3389/frai.2025.1655338

**Published:** 2025-10-31

**Authors:** Tianyu Wang, Aowen Wang, Minwei Zhu, Wenhao Jiang, Mingrui Li, Shi Yan, Yifu Shu, Shengkun Yu, Zhiguo Lin, Zhibin Han

**Affiliations:** ^1^Department of Neurosurgery, First Affiliated Hospital of Harbin Medical University, Harbin, China; ^2^Faculty of Computing, Harbin Institute of Technology, Harbin, China; ^3^Central Operating Room, Harbin Medical University Cancer Hospital, Harbin, China; ^4^Department of Myxoma, Aerospace Center Hospital, Beijing, China; ^5^Department of Neurosurgery, Fourth Affiliated Hospital of Nanjing Medical University, Nanjing, China

**Keywords:** temporal lobe epilepsy, diagnosis, biomarker, transcriptome, interpretation, Kolmogorov-Arnold Networks

## Abstract

**Introduction:**

Temporal lobe epilepsy (TLE) represents a significant neurological disorder with complex genetic underpinnings. This study aimed to develop an interpretable deep learning diagnostic model for TLE and identify disease-associated markers.

**Methods:**

Using RNA-seq and microarray data from 287 samples collected from eight GEO datasets, we constructed multiple machine learning algorithms including Deep Neural Networks (DNN), Extreme Gradient Boosting (XGBoost), Random Forest (RF), Logistic Regression (LR), and K-Nearest Neighbors (KNN) to distinguish TLE from normal. SHapley Additive exPlanations (SHAP) and Kolmogorov-Arnold Networks (KAN) were employed to interpret the model and identify key genes associated with TLE pathogenesis.

**Results:**

After comparative analysis, a Deep Neural Network (DNN) model with 10 optimized genetic features achieved perfect diagnostic performance (AUC = 1.000, accuracy = 1.000). SHAP interpretation identified DEPDC5, STXBP1, GABRG2, SLC2A1, and LGI1 as the most significant TLE-associated genes. The KAN model revealed complex nonlinear relationships between these genes and TLE status, providing mathematical expressions that capture their contributions. To facilitate clinical application, we developed an online diagnostic platform that delivers interpretable predictions based on gene expression values.

**Discussion:**

This study advances our understanding of TLE pathogenesis and provides a transparent, interpretable diagnostic model, which combines with traditional diagnostic methods may significantly improve the accuracy of TLE diagnosis, serving as a supplementary tool for clinical assessment.

## 1 Introduction

Temporal lobe epilepsy (TLE) is one of the most common forms of focal epilepsy, characterized by recurrent seizures originating from the temporal lobes of the brain, particularly the hippocampus (Thijs et al., 2019; Wang et al., 2017; Begley et al., 2022). Currently, significant advances have been made in neuroimaging and electroencephalography for clinical diagnosis. However, the molecular mechanisms underlying TLE pathogenesis remain incompletely understood (Schmidt and Schachter, 2014; Jones and Reilly, 2016; Jones and Cascino, 2016). Despite advances in antiepileptic medications, approximately 30% of TLE patients remain resistant to pharmacological treatments, necessitating surgical interventions. Nonetheless, 40–50% of individuals remain unable to attain enduring seizure freedom following surgical intervention (Stockman, 2013), and there exists a deficiency of biomarkers to predict treatment response.

This dual dilemma of timely and accurate early diagnosis and treatment response assessment highlights the urgency of developing objective assessment tools based on molecular characteristics.

Recent technological advances in high-throughput sequencing have generated vast amounts of genomic data that offer unprecedented opportunities to explore the genetic basis of neurological disorders, including epilepsy (Rosenblatt et al., 2024; Cabitza et al., 2021; Cali et al., 2022; Verhage and Sørensen, 2020). However, translating this wealth of genomic information into clinically relevant insights requires sophisticated computational approaches that can effectively model complex gene-disease relationships.

While traditional analyses of sc-seq or sn-seq data can reveal gene–disease associations, they may miss complex, non-linear interactions among genes. Machine learning, particularly deep learning algorithms, offers a key advantage in capturing such high-dimensional, non-linear patterns, has emerged as a powerful tool for analyzing high-dimensional genomic data and identifying disease-specific biomarkers (LeCun et al., 2015). Traditional machine learning models, however, often function as “black boxes,” providing predictions without revealing the underlying biological mechanisms. This lack of interpretability limits their utility in clinical settings and scientific discovery.

To address these limitations, there is growing interest in developing interpretable deep learning approaches that not only deliver accurate predictions but also provide insights into the biological mechanisms driving the predictions. Interpretable models are crucial for gaining scientific understanding and building trust in clinical applications (Lundberg and Lee, 2017; Liu et al., 2024b).

In this study, we sought to develop an interpretable deep learning-based diagnostic model for TLE using RNA-seq and microarray data. We employed five machine learning algorithms to identify the optimal approach for TLE diagnosis. Furthermore, we utilized SHAP and KAN to interpret the model and identify key genes associated with TLE pathogenesis. Our research aims to advance the molecular understanding of TLE and provide a transparent, accurate diagnostic tool with potential applications in precision medicine. By identifying key genetic drivers of TLE, we hope to contribute to the development of targeted therapies and improved patient management strategies.

## 2 Methods

### 2.1 Study design

**Figure 1:**
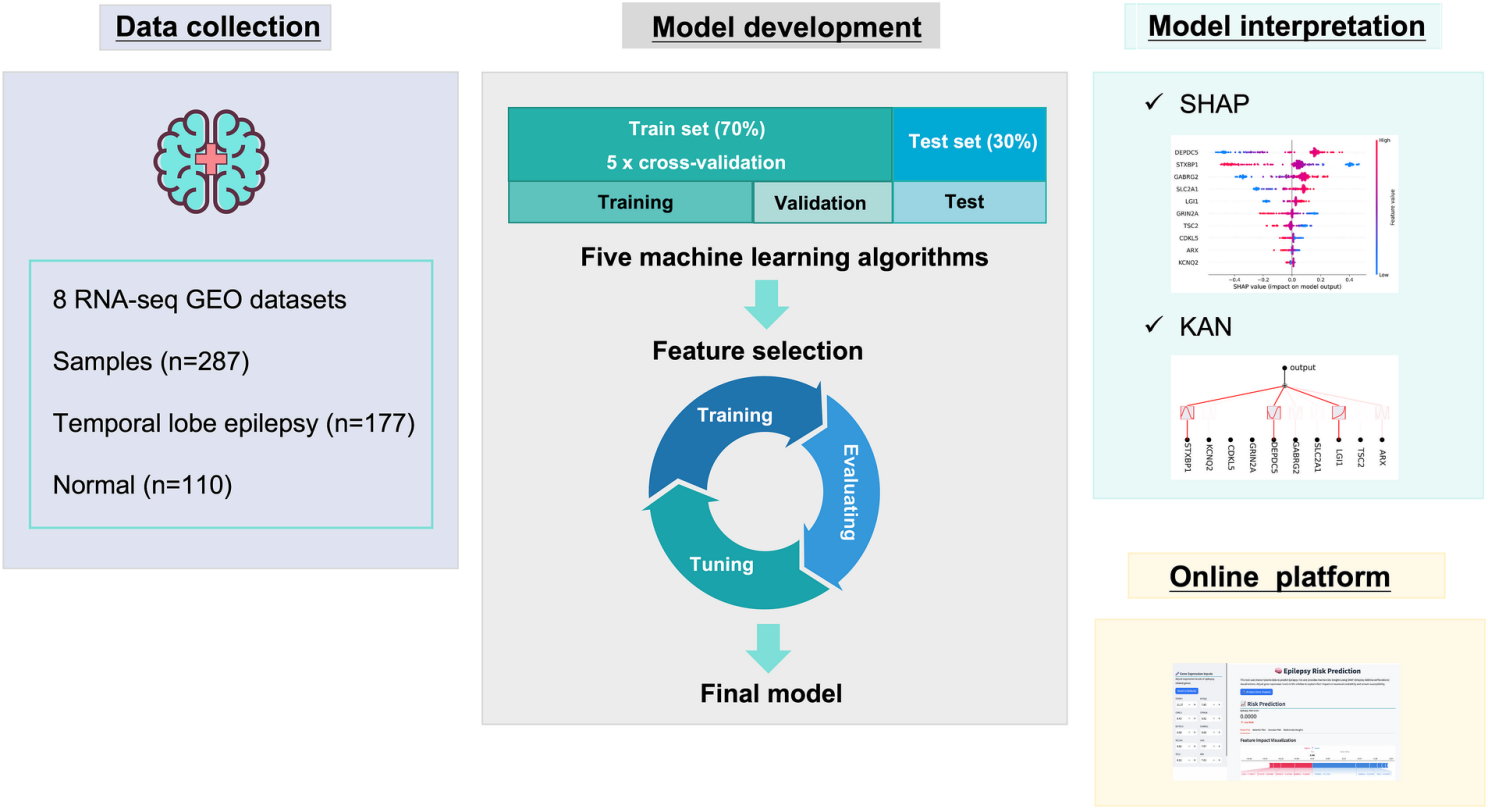
The diagram of the study design.

This research was performed as shown in Figure 1. First, data collection. Second, model development including algorithms comparisons and feature selection, the final model was determined. Next, the model interpretation was performed by SHAP and KAN. Finally, online diagnostic platform was developed.

### 2.2 Data source

We downloaded transcriptome RNA-seq and microarray data of normal temporal lobe or hippocampus and TLE patients’ hippocampus from the GEO database from 2007 to the present. A total of 287 samples were obtained, including 110 normal samples and 177 TLE samples. The datasets were mainly distributed in North America and Europe. The detailed information was shown in Table 1.

**Table 1:** Baseline characteristics of participants.

Variables	Total (*n* = 287)	Participants		*p*
		Epilepsy (*n* = 169)	Control (*n* = 118)	
GSE, *n* (%)				<0.001
GSE63808	129 (44.948)	129 (76.331)	0 (0.000)	
GSE163296	22 (7.666)	22 (13.018)	0 (0.000)	
GSE28674	18 (6.272)	18 (10.651)	0 (0.000)	
GSE44456	19 (6.620)	0 (0.000)	19 (16.102)	
GSE11882	43 (14.983)	0 (0.000)	43 (36.441)	
GSE7307	16 (5.575)	0 (0.000)	16 (13.559)	
GSE122063	22 (7.666)	0 (0.000)	22 (18.644)	
GSE104704	18 (6.272)	0 (0.000)	18 (15.254)	
Sex, *n* (%)				<0.001
Male	71 (24.739)	18 (10.651)	53 (44.915)	
Female	69 (24.042)	22 (13.018)	47 (39.831)	
Unknown	147 (51.220)	129 (76.331)	18 (15.254)	
Age_Group, *n* (%)				<0.001
20–40	10 (3.484)	0 (0.000)	10 (8.475)	
40–60	32 (11.150)	0 (0.000)	32 (27.119)	
60–80	32 (11.150)	0 (0.000)	32 (27.119)	
80–100	28 (9.756)	0 (0.000)	28 (23.729)	
Unknown	185 (64.460)	169 (100.000)	16 (13.559)	
Continents, *n* (%)				<0.001
Europe	129 (44.948)	129 (76.331)	0 (0.000)	
North America	118 (41.115)	0 (0.000)	118 (100.000)	
South America	40 (13.937)	40 (23.669)	0 (0.000)	

### 2.3 Data preprocess

To ensure comparability across datasets derived from different platforms, we conducted standardized preprocessing separately for RNA-seq and microarray data prior to integration. For RNA-seq datasets, raw expression counts were first converted to Transcripts Per Million (TPM) to account for sequencing depth and gene length. The resulting TPM matrix was then log_2_-transformed [i.e., log_2_(TPM + 1)] to stabilize variance and reduce the influence of extreme values. For microarray datasets, raw probe-level intensity values were background-corrected and quantile-normalized using platform-specific pipelines. Where multiple probes mapped to the same gene, the mean expression was taken. Only genes present across all datasets were retained. After within-platform normalization, we performed gene intersection to ensure consistent dimensions across datasets. Samples with excessive missing values or poor quality were excluded. To facilitate multi-platform integration, the RNA-seq and microarray matrices were combined into a single expression matrix, and a batch effect correction step using the ComBat algorithm (from the sva R package) was applied to remove platform-specific technical variation while preserving biological signal. Finally, we integrated multiple datasets to ensure that all samples had consistent gene dimensions and expression value ranges, providing clean and standardized input data for subsequent feature selection and model construction (Supplementary Figure 1).

### 2.4 Model construction

The integrated expression data were randomly divided into a training set (70%) and a testing set (30%). The training set was used to fit model parameters and tune hyperparameters, while the testing set was reserved to evaluate predictive performance and generalizability. Five machine learning algorithms—Deep Neural Network (DNN), Extreme Gradient Boosting (XGBoost), Random Forest (RF), Logistic Regression (LR), and K-Nearest Neighbors (KNN)—were used to construct TLE risk prediction models. For all models, five-fold cross-validation was applied on the training set to prevent overfitting. Specifically, the DNN models were trained using the Adam optimizer with cross-entropy as the loss function. Training was conducted with the following hyperparameters: Learning rate: 0.001, Batch size: 32, Number of epochs: 100, Activation function: ReLU for hidden layers, sigmoid for the output layer, Dropout rate: 0.3 to mitigate overfitting. To improve model generalization and prevent overfitting, we applied early stopping, where training was halted if the validation loss did not decrease for 10 consecutive epochs. The best-performing model (based on validation loss) was retained for downstream evaluation.

### 2.5 Feature selection

The preliminary model was constructed using a deep neural network (DNN), with input features comprising the expression values of all genes. During training, the Adam optimizer was applied with cross-entropy as the loss function. To identify the most informative features, we employed SHapley Additive exPlanations (SHAP) to evaluate feature contributions to the trained DNN model. SHAP assigns each feature a Shapley value representing its marginal contribution to model output, allowing us to rank features by their mean absolute SHAP values. Based on this ranking, we selected the top 34, top 30, and top 10 features for downstream model construction and comparison.

### 2.6 Model evaluation

Area Under the receiver operating characteristic (ROC) Curve (AUC), accuracy, precision, recall, and F1 Score were used to evaluate model performance. The calculation formulas were as follows. The four possible outcomes of classification results were true positive (TP), false positive (FP), true negative (TN), and false negative (FN).


$$\text{Accuracy}=\frac{\;\mathrm{TN}+\mathrm{TP}\;}{\;\mathrm{TP}+\mathrm{TN}+\mathrm{FP}+\mathrm{TN}\;}$$

$$\text{Precision}=\frac{\;\mathrm{TP}\;}{\;\mathrm{FP}+\mathrm{TP}\;}$$

$$\text{Recall}=\frac{\;\mathrm{TP}\;}{\;\mathrm{FN}+\mathrm{TP}\;}$$

$$F1-\text{Score}=\frac{2\times \text{Precision}\times \text{Recall}\;}{\;\text{Precision}+\text{Recall}\;}$$

### 2.7 SHapley Additive exPlanations (SHAP)

SHAP interpretation was based on the Shapley value in game theory, offering explanations on both global and local levels by assessing the incremental contribution of each feature to the model’s predictive outcomes across various samples (Lundberg and Lee, 2017). SHAP interpretation was based on Shapley values from cooperative game theory, providing both global and local interpretability by quantifying the marginal contribution of each feature to the model’s prediction across different samples. At the global level, feature importance was assessed using two primary visualizations: bar plots of mean absolute SHAP values to rank features by their overall contribution, and Beeswarm plots to display the distribution of SHAP values for each feature across the entire dataset, capturing both the importance and directionality of their effects. In addition, dependence plots were used to show how the SHAP value of a given feature changed with its actual value, thereby highlighting potential nonlinear or interaction effects. At the local level, we employed force plots and waterfall diagrams to interpret predictions for individual samples. Force plots visually illustrated how each feature pushed the model output toward or away from a particular classification, while waterfall diagrams provided a step-by-step breakdown of how the cumulative SHAP values of all features contributed to the final prediction score for a single instance. This multi-level interpretability framework allowed us not only to identify the most influential genes contributing to the diagnostic outcome but also to trace their sample-specific effects, thereby enhancing the transparency, biological plausibility, and clinical credibility of the model.

### 2.8 Kolmogorov-Arnold Networks (KAN)

The KAN model was established based on the Kolmogorov-Arnold representation theorem, possessing the ability to output explicit expressions in functional (Liu et al., 2024b; Xu et al., 2024). KAN could construct regularization and grid sparsity optimization, outputting interpretable mathematical forms that clearly reveal the mapping relationship between key input features and model predictions. By analyzing the parameter distribution and activation patterns in the KAN network, key gene features could be identified that influence prediction results, thereby assisting in the inference and validation of biological mechanisms.

To visualize the internal architecture and interpret the learned representations, network topology plots were generated, displaying the organization of hidden nodes and their activation patterns. For each selected feature, the corresponding functional mapping was extracted directly from the trained KAN and expressed as the explicit mathematical formula [e.g., f(x) = a + bx], which was further illustrated through fitted curve plots with *R*^2^ statistics to reflect the quality of the approximation. In addition, sample-specific importance analysis was conducted to reveal the individualized contribution of each gene to model predictions. By computing local activation values at key nodes, the KAN provided insight into which genes most strongly promoted or inhibited disease classification on a per-sample basis. This enabled biologically meaningful interpretation at both the population and individual levels.

### 2.9 Online computing platform

The web application of the TLE risk prediction model was developed based on the Streamlit framework.

### 2.10 Statistical analysis

All data analyses were performed using R version 4.2.2 and Python version 3.9.12, and all statistical tests were conducted using two-sided tests, with *p* < 0.05 considered statistically significant.

## 3 Results

### 3.1 Patients characteristics

This study included a total of 287 samples, with 169 from epilepsy cohort and 118 from normal control cohort. The detailed information was shown in Table 1. The epilepsy samples were mainly from GSE63808 (76.331%), while the normal control samples were primarily distributed in GSE11882 (36.441%) and GSE104704 (15.254%).

### 3.2 Model development

**Figure 2:**
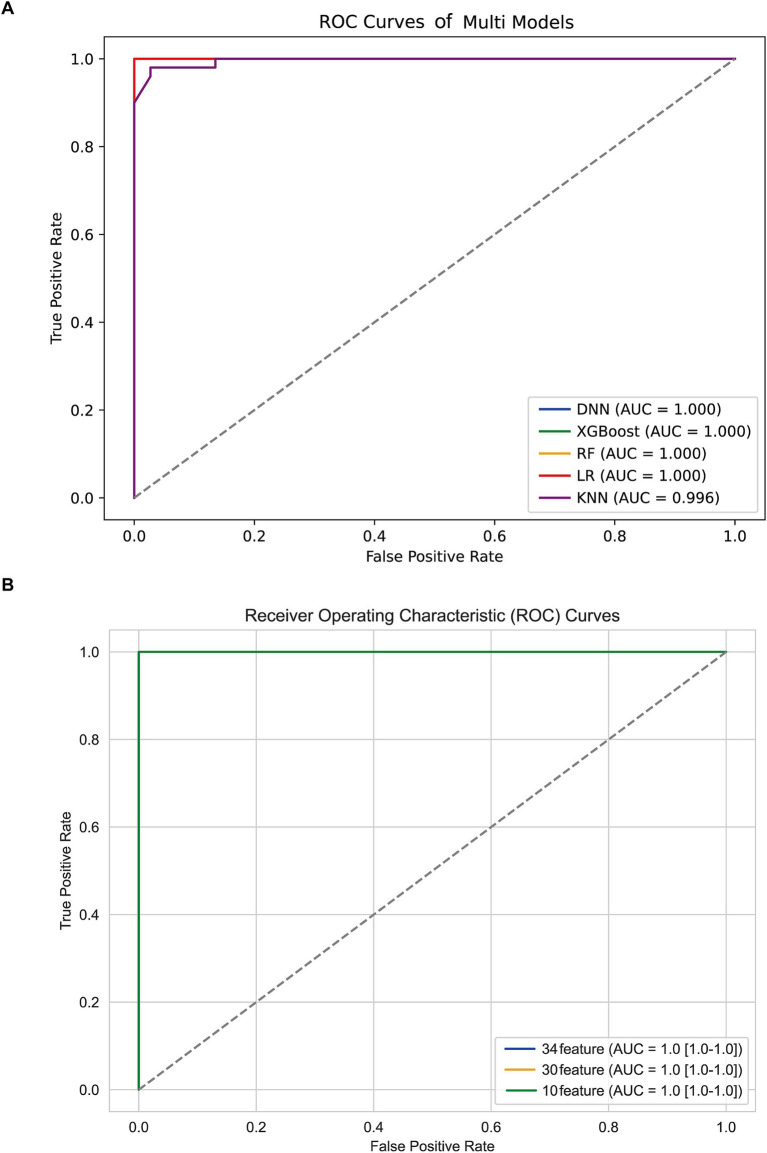
ROC curves **(A)** different algorithms **(B)** DNN models with different features.

All the TLE-related features were used to train five machine learning algorithms and the diagnostic performance was evaluated and compared as shown in Figure 2A; Table 2. All algorithms except KNN exhibited an excellent predictive performance with the AUC of 1.000, the accuracy, precision, recall and F1-socre of 1.000. The predictive capability of KNN was commendable as well, with the AUC of 0.996, the accuracy of 0.977, the precision, recall and F1-socre of 0.980. These results suggest that these features held considerable significance, as they exhibited robust predictive capabilities across various algorithms. DNN was randomly selected for the further analysis based on the excellent performance of all algorithms.

**Table 2:** Model performance of multiple algorithms for epilepsy diagnosis.

Model	AUC	Accuracy	Precision	Recall	F1 score
DNN	1.000	1.000	1.000	1.000	1.000
XGBoost	1.000	1.000	1.000	1.000	1.000
RF	1.000	1.000	1.000	1.000	1.000
LR	1.000	1.000	1.000	1.000	1.000
KNN	0.996	0.977	0.980	0.980	0.980

### 3.3 Feature optimization

To reduce model complexity and improve model prediction performance, feature selection was conducted based on 34, 30, and 10 features, respectively. As shown in Figure 2B; Table 3, the model performance in the test data was also superior with the AUC, accuracy, precision, recall and F1-score of 1.000 among the DNN model with different features. The predictive performance of DNN model with 10 features in train data was shown in Supplementary Figure 2. Thus, the DNN model with 10 features was identified as the final model in this study.

**Table 3:** Predictive performance of models with different features.

Model	AUC (95%CI)	Accuracy	Precision	Recall	F1 score
34 feature	1.000 (1.000, 1.000)	1.000	1.000	1.000	1.000
30 feature	1.000 (1.000, 1.000)	1.000	1.000	1.000	1.000
10 feature	1.000 (1.000, 1.000)	1.000	1.000	1.000	1.000

To evaluate the potential overfitting risk due to sample imbalance, we performed a sensitivity analysis by excluding GSE63808 from the training set. The model was retrained and tested on the remaining datasets. Remarkably, the diagnostic model maintained excellent performance with an AUC of 1.00 (Supplementary Figure 3), demonstrating its robustness and generalizability across independent cohorts.

### 3.4 Model interpretation

#### 3.4.1 SHAP analysis

**Figure 3:**
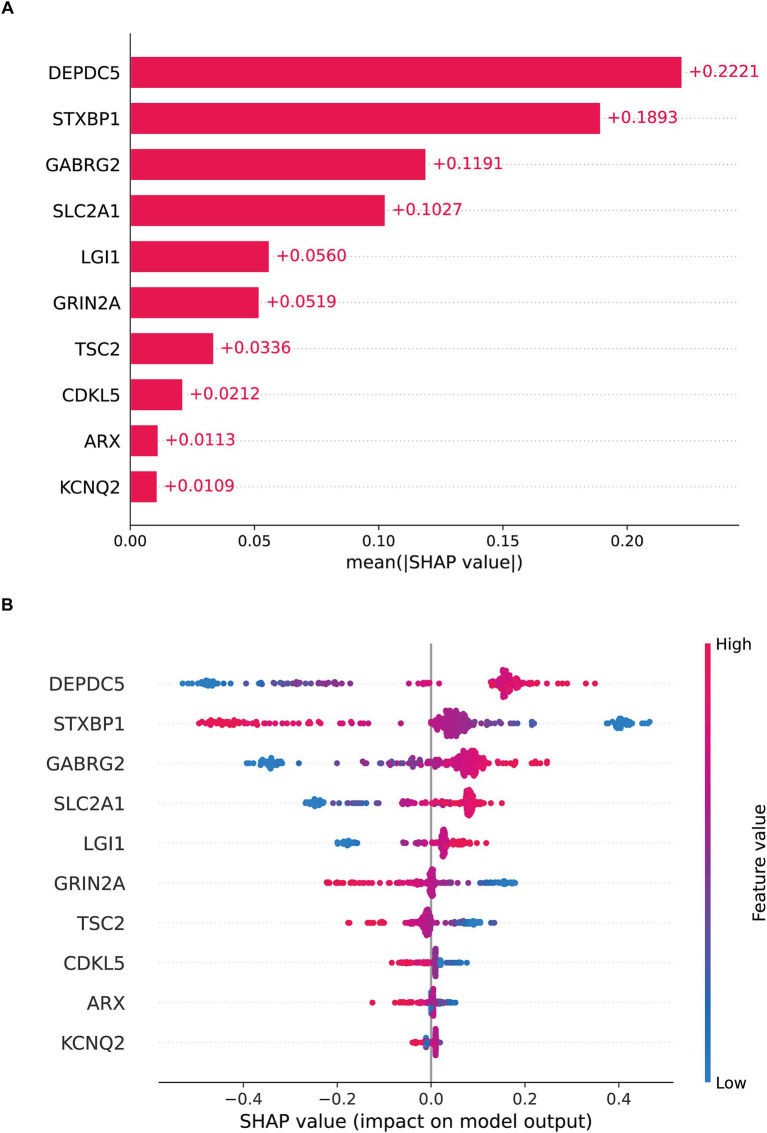
SHAP global interpretation **(A)** the Bar diagram **(B)** the Beeswarm plot.

**Figure 4:**
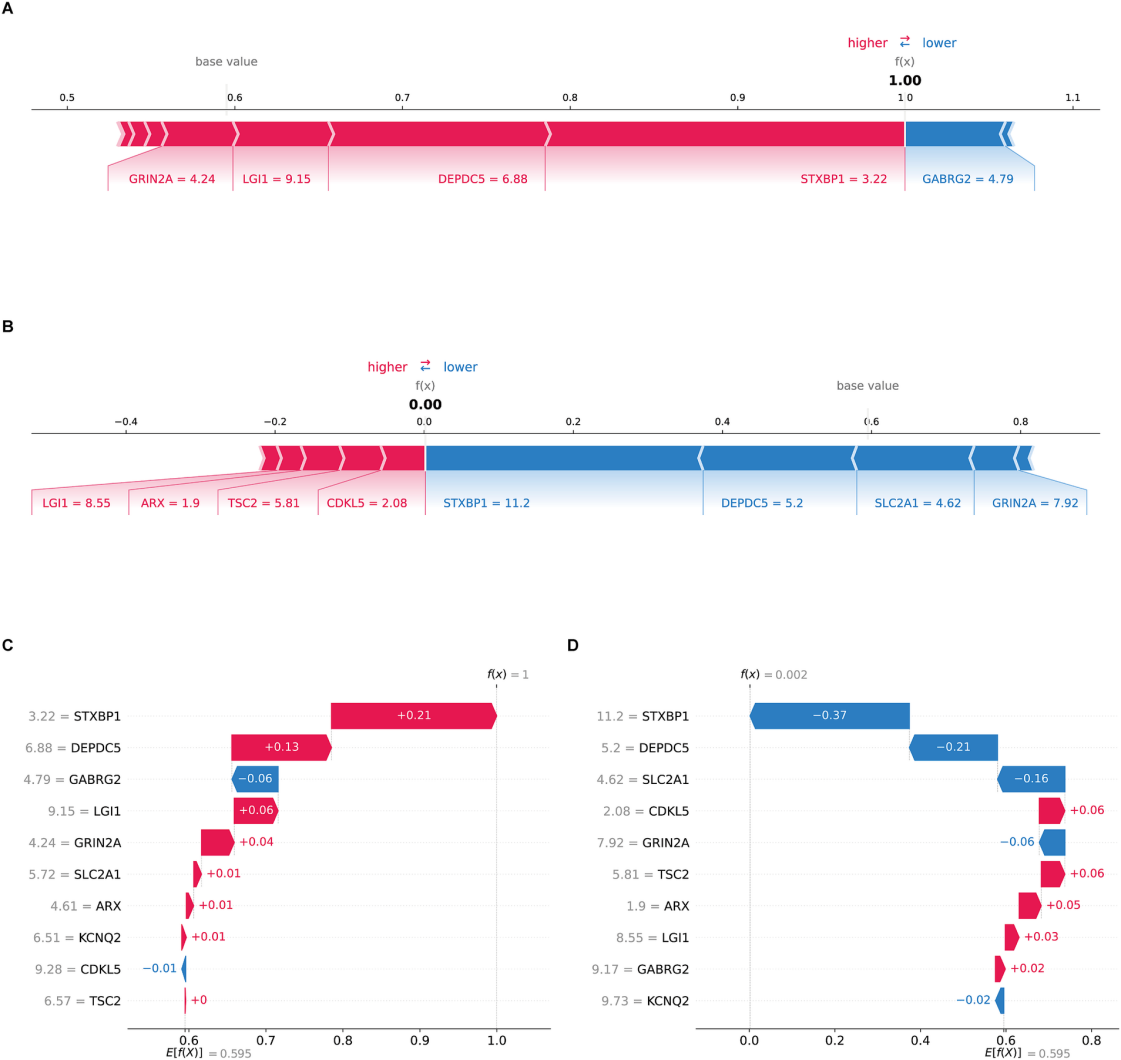
SHAP local interpretation **(A)** the f(x) value of the force plot was 1.00, indicating the patient was TLE. GRIN2A, LGI1, DEPDC5, and STXBP1 (red arrows) are important drivers of elevated predictive values, while GABRG2 (blue arrow) exerts a negative effect. **(B)** The f(x) of the force plot was 0.00, indicating the patient was normal. **(C)** The waterfall diagram showed the Ef(x) was 0.595, with the positive contributions of STXBP1 (+0.21), DEPDC5 (+0.13), LGI1 (+0.06) and GRIN2A (+0.04) and the negative contributions of GABRG2 (−0.06), push the predictive result to TLE as f(x) was 1. **(D)** The waterfall diagram presented a diagnostic result of normal. The baseline forecast value Ef(x) was 0.595, with the negative contributions of STXBP1 (−0.37), DEPDC5 (−0.21), SLC2A1 (−0.16) and GRIN2A (−0.06) and the positive contributions of CDKL5 (+0.06), TSC2 (+0.06), the final f(x) was 0.002.

SHAP was employed to enhance model interpretability. As shown in Figure 3A, the most important pathogenic genes related to TLE were DEPDC5, STXBP1, GABRG2, SLC2A1, LGI1, GRIN2A, TSC2, CDKL5, ARX, KCNQ2. The higher levels of DEPDC5 increased the risk of TLE (Figure 3B). Figures 4A,B visualized the contributions of features to the diagnostic result for individual patients (TLE or normal). Figure 3A displayed a diagnostic outcome for TLE, as GRIN2A, LGI1, DEPDC5, and STXBP1 were important drivers for increasing the predicted value, while GABRG2 exerted a negative effect. Figure 3B showed a predictive result for normal, with STXBP1, DEPDC5, ARX, and CDKL5 significantly inhibiting the prediction tendency for epilepsy, while LGI1 and GABRG2 slightly increased the predicted value. Figures 4C,D displayed the decision-making process of multiple features on prediction outcome. The interplay of diverse features pushed the diagnostic outcome to TLE or normal. In addition, the scatter analysis further revealed the relationship between SHAP values and key features (Supplementary Figure 4). STXBP1, CDKL5, GRIN2A, TSC2, and ARX made negative contributions to SHAP values, while DEPDC5, GABRG2, SLC2A1, LGI1 contributed positively to SHAP values.

#### 3.4.2 KAN optimization

**Figure 5:**
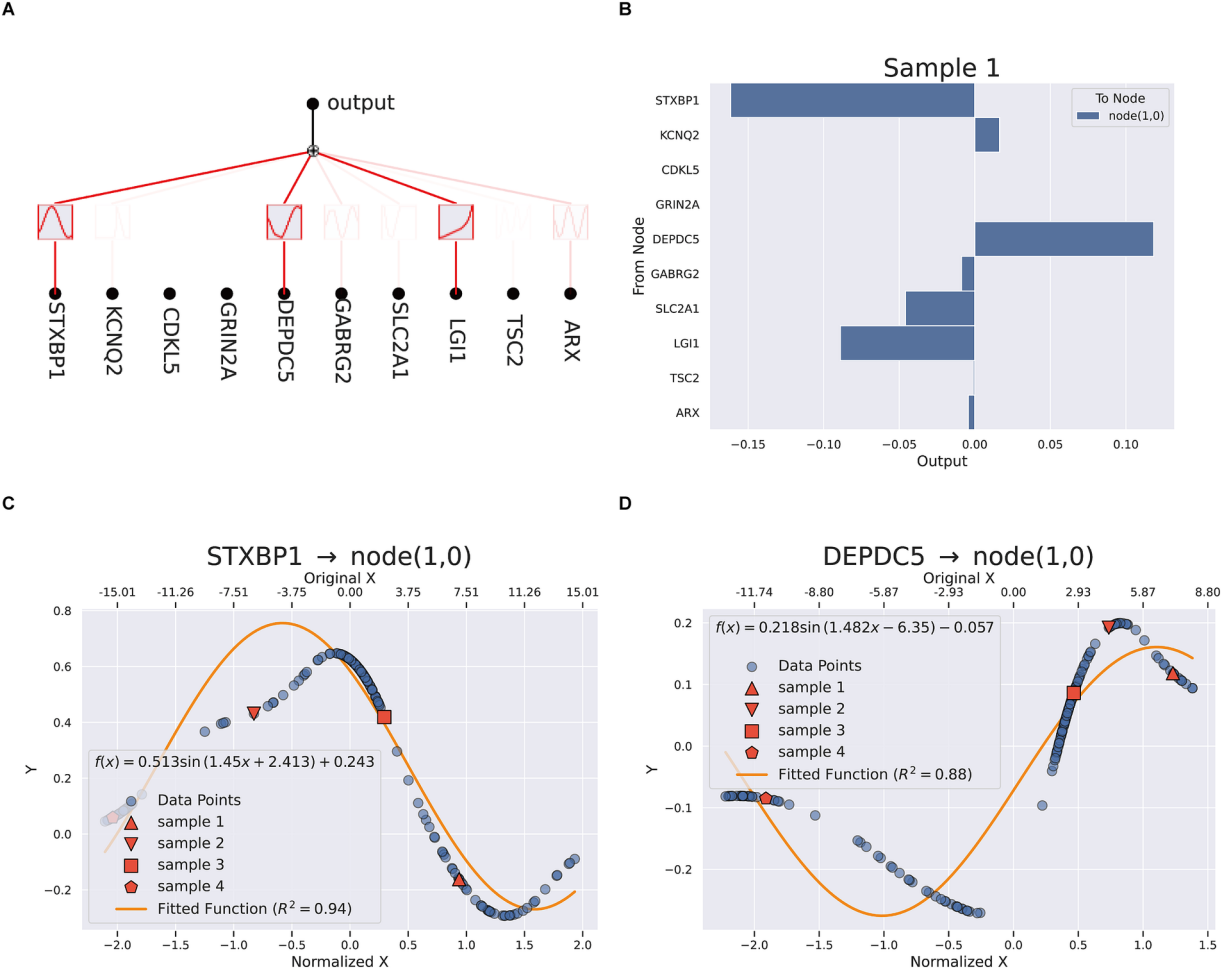
KAN interpretation **(A)** the network analysis **(B)** feature importance in sample 1. **(C,D)** Fitting equation between STXBP1, DEPDC5 and node (1,0).

To further improve the interpretability and the transparency of the model, the final model was explained and optimized by KAN. Figure 5A illustrated the complex and nonlinear contributions of essential features. A total of ten genes including STXBP1, KCNQ2, CDKL5, GRIN2A, DEPDC5, GABRG2, SLC2A1, LGI1, TSC2, and ARX, were utilized as input nodes. Each of these input features was connected to intermediate nodes through various nonlinear functions (such as sine and logarithm) and aggregated through linear combinations or activation functions across multiple pathways, ultimately yielding the anticipated outcomes. STXBP1, DEPDC5, and LGI1 contributed significantly to the output, indicating their key driving role in the classification task. The mathematical expression of the KAN model was shown in Supplementary Figure 5, which was composed of a weighted combination of multiple input features after nonlinear transformations. Figure 4B provided a quantitative evaluation of the individual contributions of each feature to the model’s predictive results. The findings revealed that STXBP1 and DEPDC5 exerted a pronounced influence on the predictive outcomes, whereas ARX and TSC2 made a lesser impact, indicating their restricted discriminative capacity within the current model framework. Figures 4C,D illustrated the nonlinear mapping relationship between the characteristic values of STXBP1 and DEPDC5 along with their intermediary nodes. The correspondence of STXBP1 to node (1,0) demonstrated an excellent fit, with a curve *R*^2^ for 0.94, which indicated that the nonlinear transformation effectively captured the response mechanism linking its characteristic alterations to network outputs. Likewise, the mapping relationship of DEPDC5 also revealed robust nonlinear fitting (*R*^2^ = 0.88), further validating the superiority of the KAN model in handling intricate input features. Additionally, the pathway enrichment analysis of key genes related to TLE was mainly in neuropeptide hormone activity, neuropeptide receptor binding, lumenal side of membrane, transport vesicle (Supplementary Figures 6–8).

### 3.5 Online diagnostic platform

**Figure 6:**
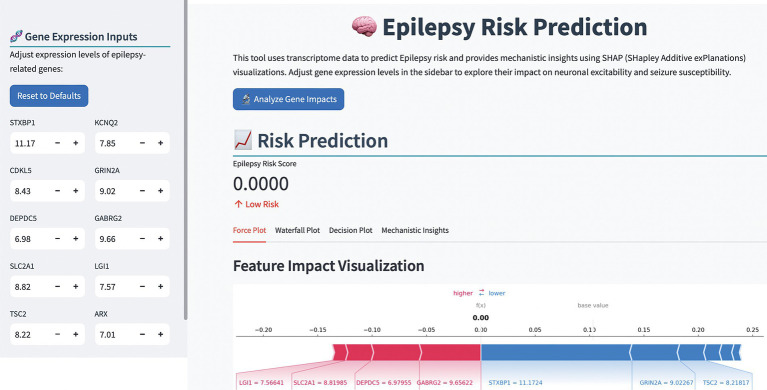
The representative image of the TLE risk prediction platform.

To enhance the clinical application of the model, we developed an online computer platform.^1^ As shown in Figure 6, the values of the TLE-related genes were entered on the left of the web page, the predictive result would appear on the right along with the SHAP explanation, which provided a visualizations of feature impacts and a mechanistic insight into TLE pathogenesis.

## 4 Discussions

Although TLE has relatively characteristic clinical manifestations, its diagnosis still faces challenges. It was found that about 30–40% of TLE patients may not show obvious abnormalities in routine electroencephalogram (EEG) examinations, leading to diagnostic delays and inappropriate treatment (Bernasconi et al., 2011). In this study, we developed a highly accurate diagnostic model for TLE using interpretable deep learning approaches. By integrating multiple RNA-seq and microarray data and employing feature optimization, we identified a set of 10 key genes that demonstrate exceptional discriminative power between TLE and normal. The perfect diagnostic performance achieved by our model (AUC = 1.000, accuracy = 1.000) across different algorithms highlights the robustness of these genetic markers as diagnostic indicators for TLE. Our research indicates that combining molecular biomarkers with traditional diagnostic methods may significantly improve the accuracy of TLE diagnosis. The deep learning model we developed based on 10 key genes can serve as a supplementary tool for clinical assessment, especially in cases where routine imaging examinations are negative, or results are uncertain.

To further validate the robustness of our DNN model, we conducted architectural ablation studies by systematically modifying the number of hidden layers, units per layer, and activation functions. As shown in Supplementary Table 1, the model maintained a perfect AUC (1.000) under most architecture variants, demonstrating remarkable structural stability. However, removing input normalization (A4) led to a notable drop in validation AUC (from 1.000 to 0.822), highlighting the critical role of normalization in preserving model performance. These findings support the soundness of our default architecture design and preprocessing pipeline.

The application of interpretability techniques, specifically SHAP and KAN, provided valuable insights into the biological mechanisms underlying our model’s predictions. SHAP analysis revealed that DEPDC5, STXBP1, GABRG2, SLC2A1, and LGI1 were the most influential genes in the diagnostic model. These findings align with previous research implicating these genes in epilepsy pathogenesis (Kang and MacDonald, 2016; Li et al., 2024; Shen et al., 2017; Larsen et al., 2015; Suls et al., 2008; Boillot et al., 2014; Dubey et al., 2020; Stafstrom, 2025). For instance, DEPDC5 mutations have been associated with various focal epilepsies, including TLE, through dysregulation of the mTOR pathway, which controls neuronal growth and excitability (Liu et al., 2025; Hughes et al., 2017). Similarly, STXBP1 plays a crucial role in synaptic vesicle docking and fusion, and its dysfunction has been linked to early-onset epileptic encephalopathies (Stamberger et al., 2022, 2023; Mignot et al., 2011).

The KAN model further enhanced our understanding by providing explicit mathematical expressions that capture the nonlinear relationships between gene expression patterns and TLE status. The strong fitting curves (*R*^2^ = 0.94 for STXBP1 and *R*^2^ = 0.88 for DEPDC5) demonstrate that KAN effectively modeled the complex interactions between these genes and the disease phenotype. This mathematical transparency represents a significant advancement over traditional “black box” neural networks, offering mechanistic insights that could inform targeted therapeutic strategies (Liu et al., 2024a,b; Hou et al., 2024).

Pathway enrichment analysis of our identified genes revealed significant associations with neuropeptide hormone activity, neuropeptide receptor binding, membrane functions, and transport vesicles. These biological processes are critical for maintaining neuronal homeostasis and synaptic transmission (Strand et al., 1991; Hirokawa and Takemura, 2005) further supporting the biological relevance of our findings. Disruptions in these pathways could contribute to the hyperexcitability and abnormal neuronal synchronization characteristic of epileptic seizures.

The development of an online diagnostic platform represents a practical translation of our research findings. This user-friendly tool allows clinicians to input gene expression values and receive instant, interpretable predictions regarding TLE risk. The incorporation of SHAP visualizations in the platform enhances its utility by providing transparent explanations for each prediction, potentially facilitating clinical decision-making and patient communication.

An important clinical distinction in temporal lobe epilepsy (TLE) lies between patients with and without hippocampal sclerosis (HS), as these subtypes may differ in etiology, pathology, and treatment response. However, this distinction was not addressed in our study due to the lack of HS-specific annotations in the publicly available datasets used. As a result, we were unable to perform stratified analyses to explore potential molecular differences between HS and non-HS subtypes. Future work incorporating high-resolution imaging data or histopathological labels will be critical to disentangle subtype-specific gene expression patterns and further refine diagnostic models. Including such data may also improve the model’s applicability in precision medicine settings.

Despite the promising performance of our model, certain limitations should be acknowledged. First, a large proportion (76%) of TLE samples in our dataset were derived from a single study (GSE63808), which may introduce potential bias or overfitting in the training process. To assess the impact of this imbalance, we performed a sensitivity analysis by excluding GSE63808 from the training data and re-evaluated model performance. Notably, the model retained excellent discriminatory ability, with an AUC of 1.0 (Supplementary Figure 3), thereby supporting its robustness across independent datasets. Nevertheless, we recognize that external validation using completely independent cohorts—ideally with more balanced representation and additional clinical annotations—is essential for further verifying the model’s generalizability and translational utility.

Another key limitation of this study is the lack of Asian patient data. Most of the included samples were from Euro-American populations, potentially limiting the model’s generalizability to other ethnic groups. Population-specific genetic and environmental factors may influence gene expression and disease risk, and thus should not be overlooked. Finally, while our interpretability approaches provide valuable insights, the complex interplay between genetic, environmental, and epigenetic factors in TLE pathogenesis requires further investigation.

Future research directions include validating our findings in prospective clinical studies, expanding the model to incorporate additional data modalities (such as neuroimaging and clinical variables), and exploring the potential of our identified genes as therapeutic targets. Additionally, investigating the longitudinal changes in gene expression patterns throughout disease progression could enhance our understanding of TLE pathogenesis and potentially enable early intervention strategies.

In conclusion, our study demonstrates the power of interpretable deep learning approaches in advancing our understanding of TLE pathogenesis and improving diagnostic capabilities. By identifying key genetic markers and elucidating their functional relationships, we have contributed to the growing body of knowledge on the molecular basis of epilepsy. The developed diagnostic platform offers a promising tool for clinical application, potentially facilitating personalized medicine approaches in epilepsy management.

## Data Availability

Publicly available datasets were analyzed in this study. This data can be found here: The gene expression datasets analyzed in this study are available in the Gene Expression Omnibus (GEO) repository at https://www.ncbi.nlm.nih.gov/geo/ under accession numbers GSE11882, GSE28674, GSE44456, GSE63808, GSE163296, GSE7307, GSE104704, and GSE122063. All other data supporting the findings are included in the manuscript or Supplementary materials.

## Footnotes

^1^ https://epilepsy2.streamlit.app
