# Supplementary material for: Construction of a diagnostic model for temporal lobe epilepsy using interpretable deep learning: disease-associated markers identification
Source: Front Artif Intell. 2025 Oct 31;8:1655338. doi: 10.3389/frai.2025.1655338 (PMC12615413; doi:10.3389/frai.2025.1655338)
Supplement: Supplementary file 1 [file Data_Sheet_1.docx]

**Supplementary Materials**

contents

Supplementary Figure 1. Batch effect correction and Principal component analysis (PCA) between epilepsy and normal control.

Supplementary Figure 2. The ROC curve of DNN model with 10 features in train set.

Supplementary Figure 3. ROC curve excluding GSE63808.

Supplementary Figure 4. The scattor plots between SHAP values and the key genes.

Supplementary Figure 5. The mathematical expression formula of KAN

Supplementary Figure 6. GO analysis of key genes in biological process, cellular component, and molecular function.

Supplementary Figure 7. KEGG analysis of key genes

Supplementary Figure 8. GSEA analysis of key genes

Supplementary Table 1. DNN Architecture ablation analysis


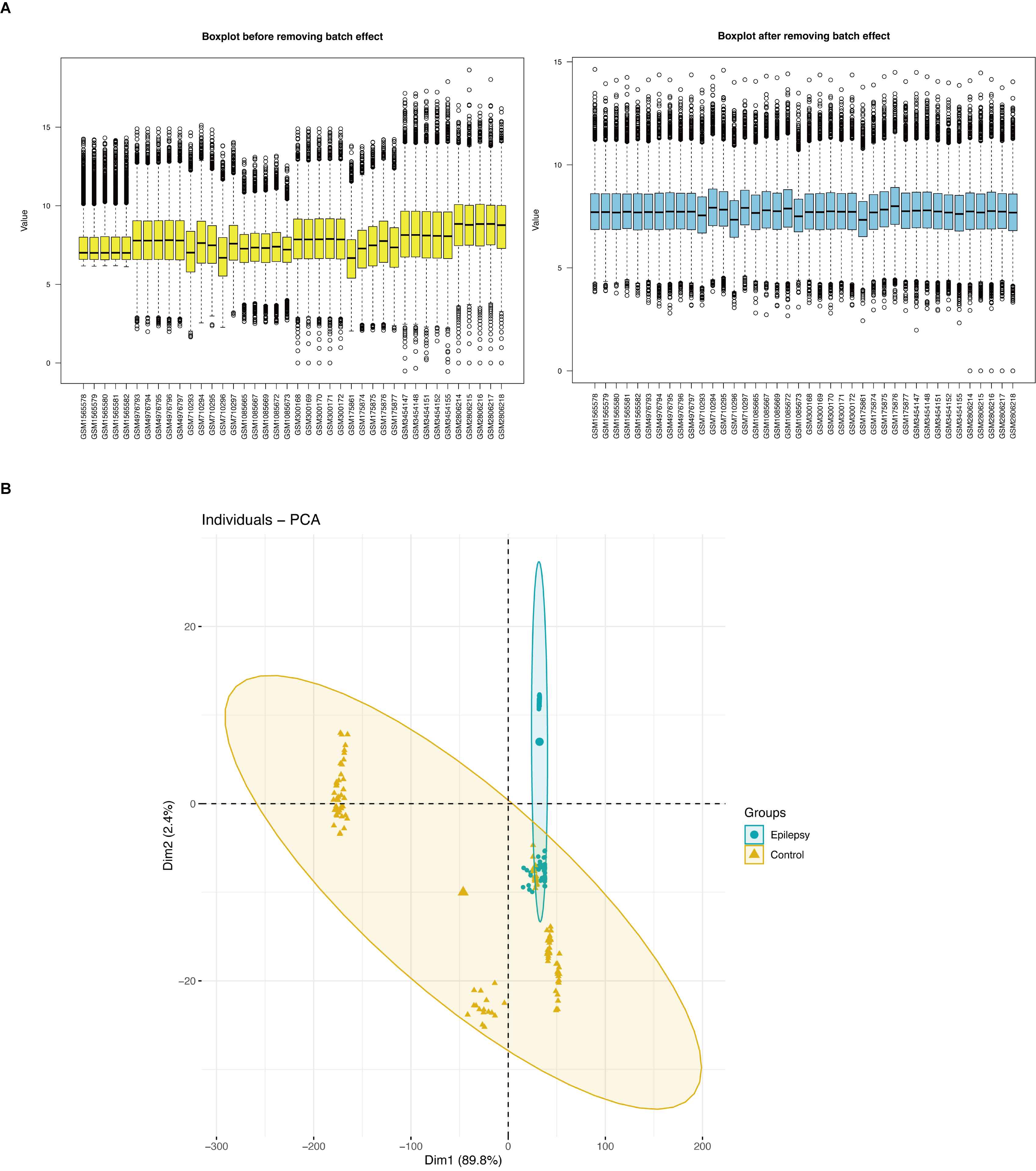


Supplementary Figure 1. Batch effect correction and Principal component analysis (PCA) between epilepsy and normal control. (A) Boxplots showing the distribution of expression values across batches before and after batch correction. Correction reduced inter-batch variability while preserving biological signal. (B) PCA was performed on the integrated data after batch effect correction to evaluate the effectiveness of normalization. The samples no longer clustered by batch, indicating that batch-associated variations were substantially mitigated while preserving underlying biological signals.


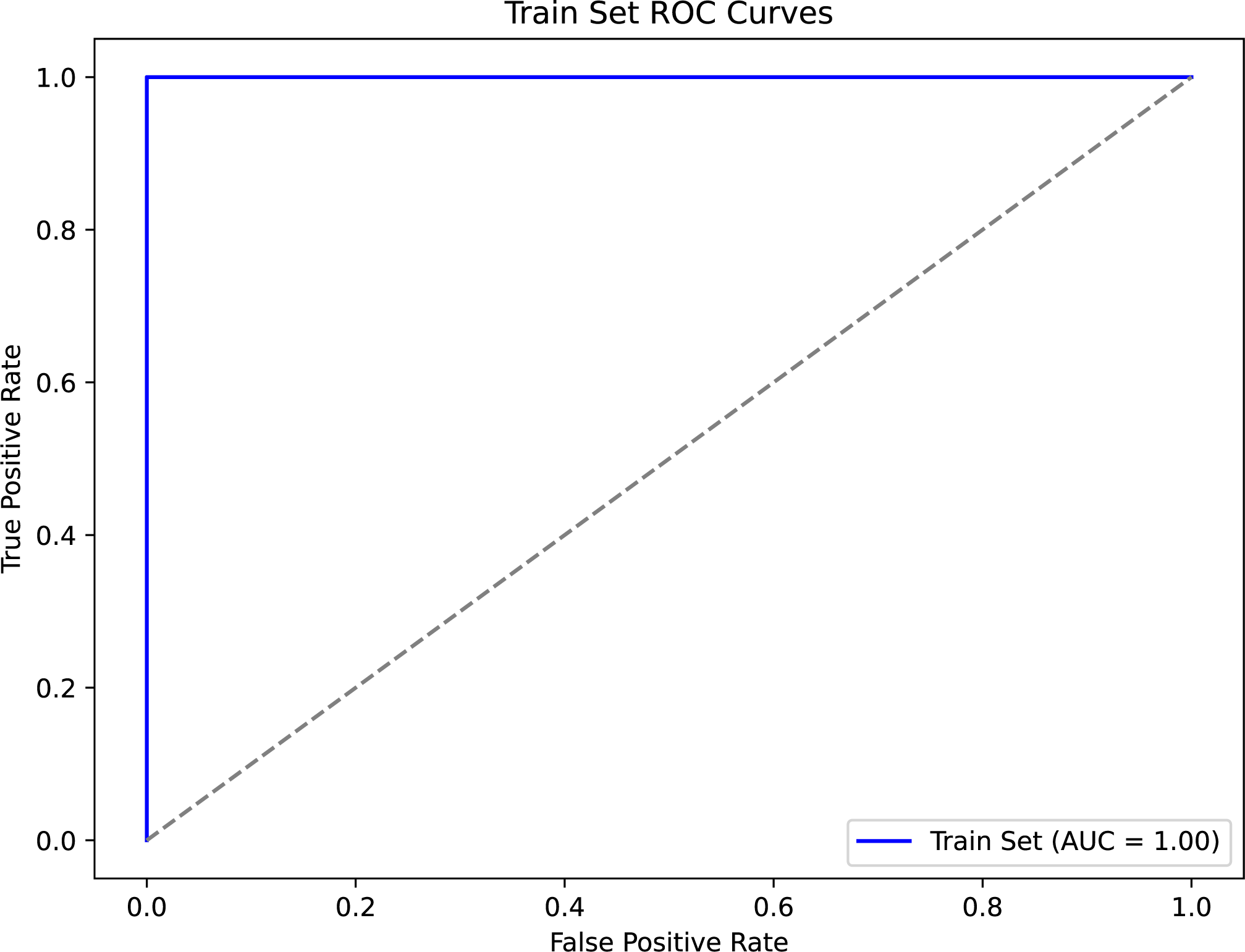


Supplementary Figure 2. The ROC curve of DNN model with 10 features in train set.


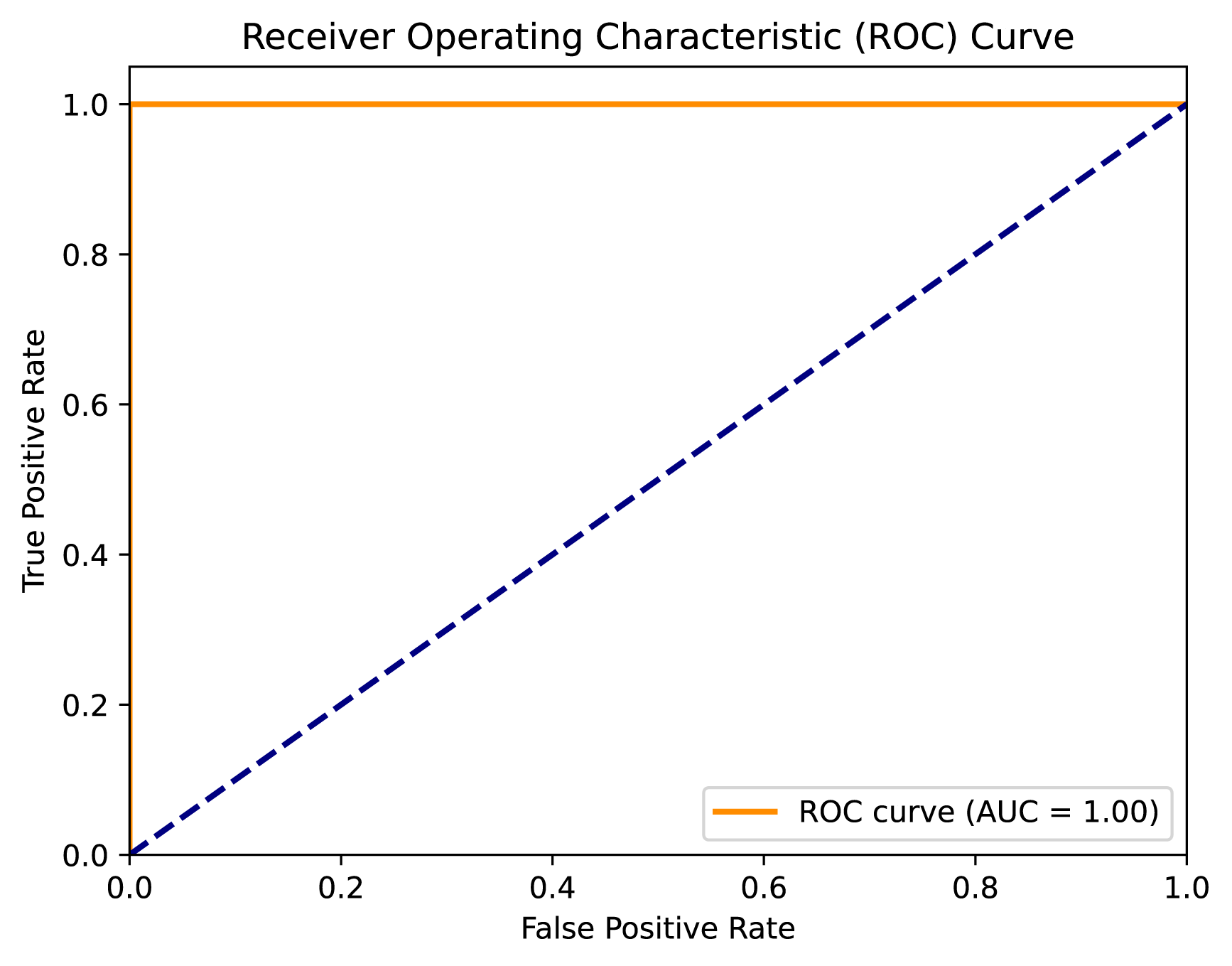


Supplementary Figure 3. ROC curve excluding GSE63808.


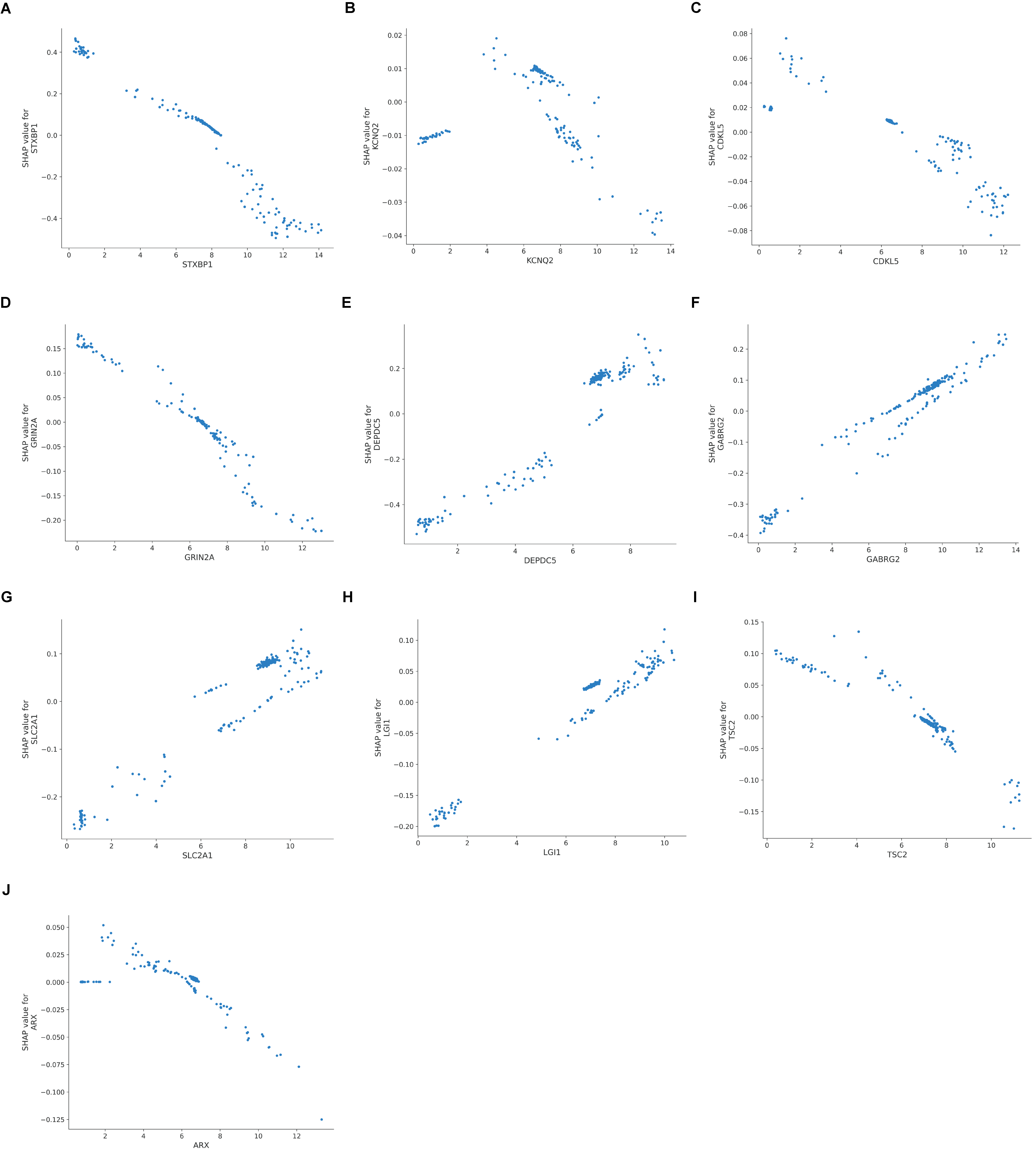


Supplementary Figure 4. The scattor plots between SHAP values and the key genes.


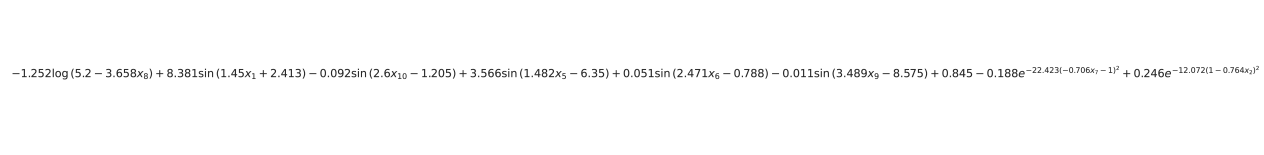


Supplementary Figure 5 The mathematical expression formula of KAN


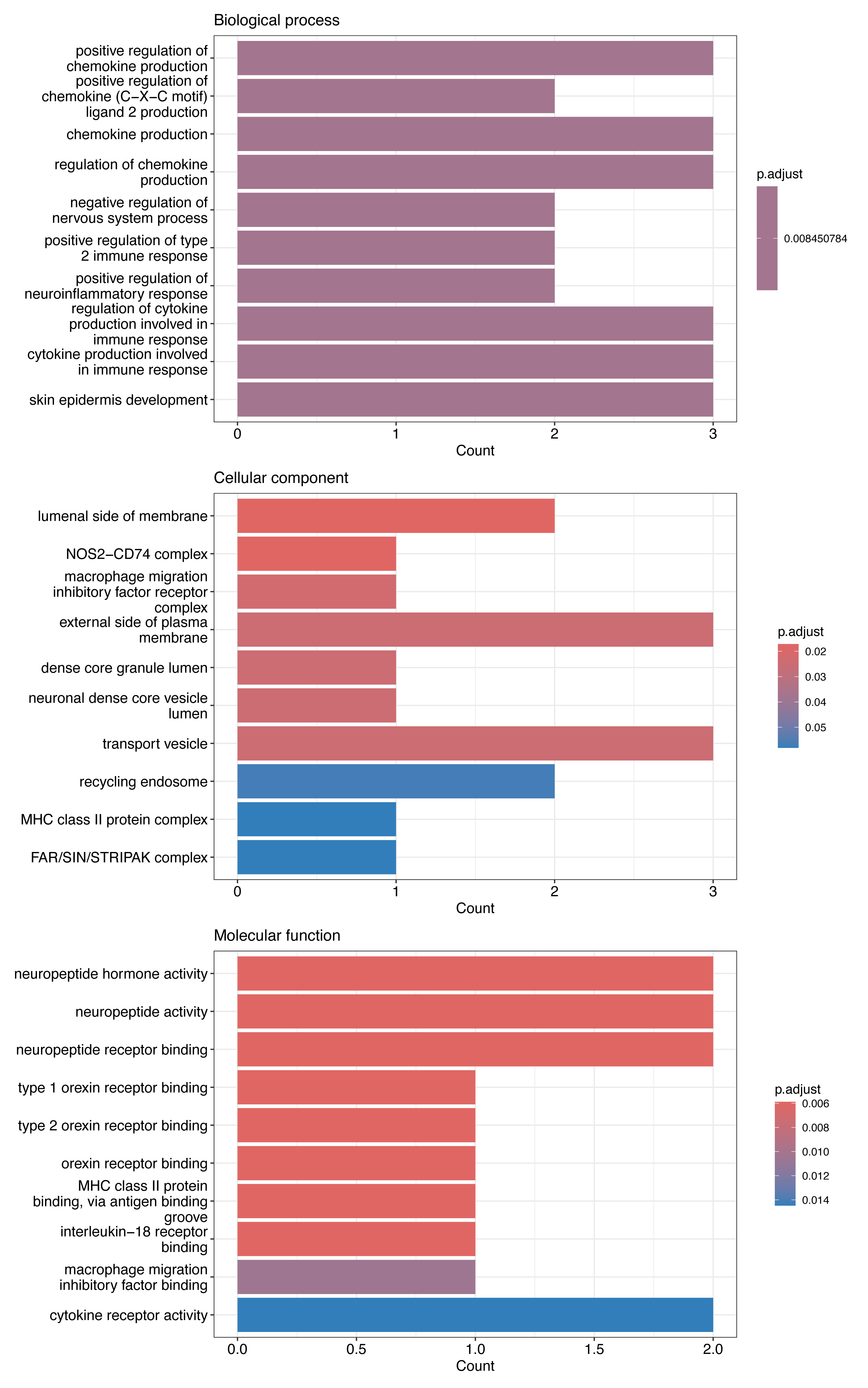


Supplementary Figure 6. GO analysis of key genes in biological process, cellular component, and molecular function.


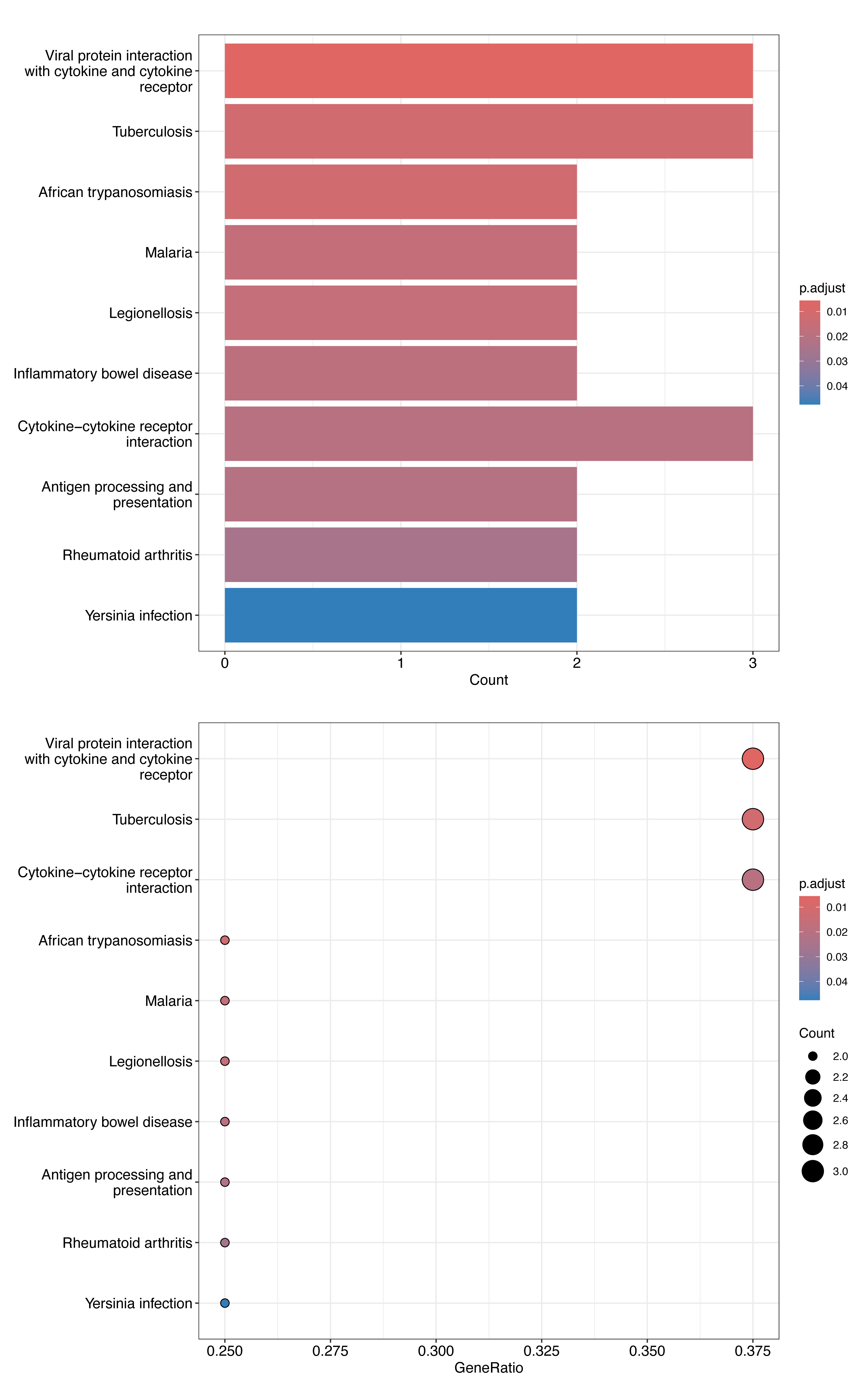


Supplementary Figure 7. KEGG analysis of key genes.


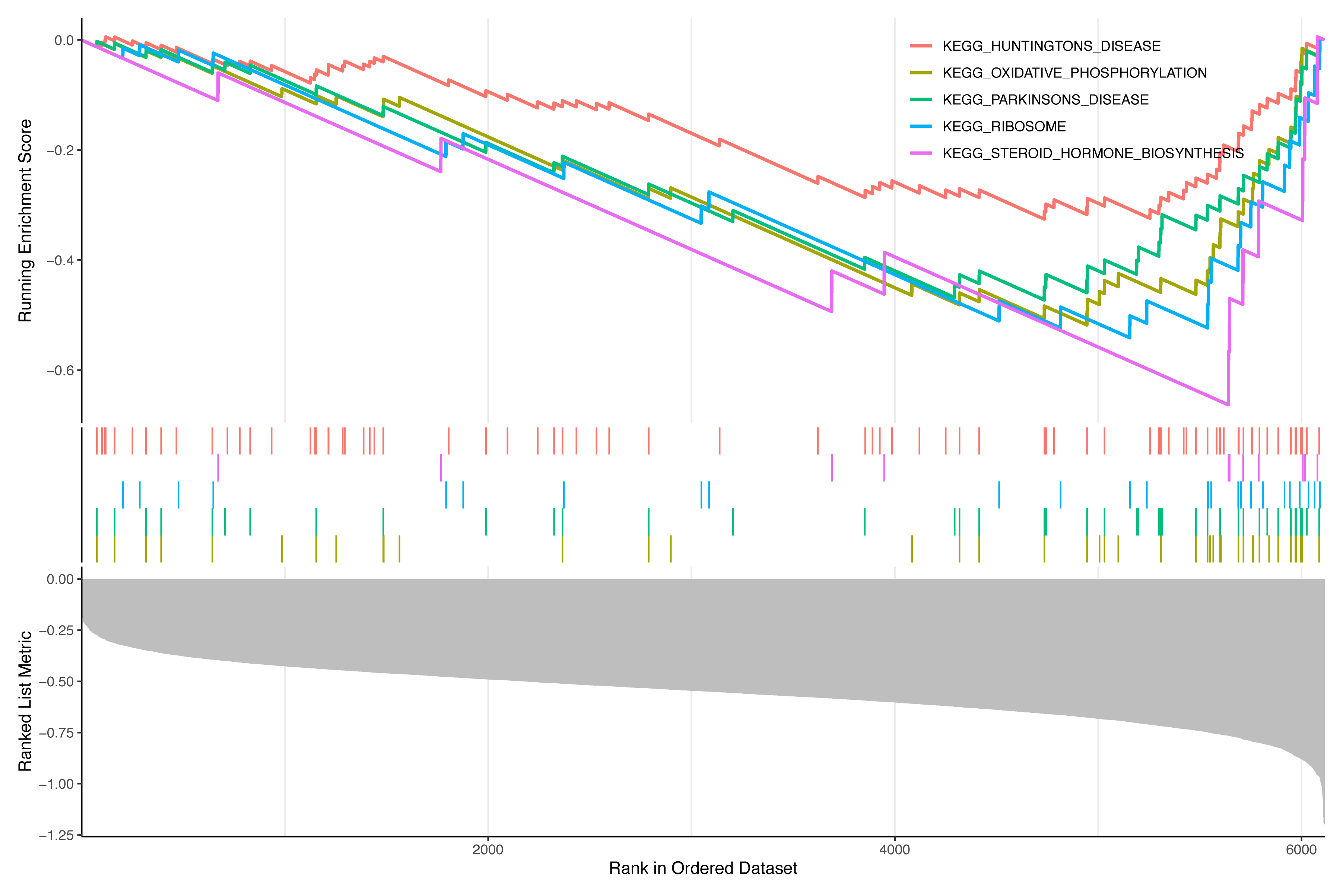


Supplementary Figure 8. GSEA analysis of key genes.

Supplementary Table 1. DNN Architecture ablation analysis.

| Architecture Variant | Hidden Layers | Units | Train_AUC | Val_AUC |
| --- | --- | --- | --- | --- |
| Baseline | 3 | 64 | 1.000 | 1.000 |
| A1 (remove HL) | 1 | 64 | 1.000 | 1.000 |
| A2 (32-16 units) | 3 | 32-16 | 1.000 | 1.000 |
| A3 (Tanh activation) | 3 | 64 | 1.000 | 1.000 |
| A4 (with no inputNorm) | 3 | 64 | 0.852 | 0.822 |
